# Outcomes and guideline adherence for emergency department thoracotomy: a single-center experience in Saudi Arabia

**DOI:** 10.1186/s12245-025-00992-3

**Published:** 2025-10-09

**Authors:** Mohammed Alageel, Tareq Al-Salamah, Latifa Alshabib, Yara Al-digi

**Affiliations:** 1 Department of Emergency Medicine and Critical Care, College of Medicine, King Saud University, Riyadh, Saudi Arabia; 2 Department of Emergency Medicine,, University of British Columbia Vancouver, Canada University, Riyadh, Saudi Arabia; 3 Department of Emergency Medicine, King Saud University Medical City, King Saud University, Riyadh, Saudi Arabia

**Keywords:** Emergency department, Resuscitative thoracotomy, Saudi arabia, traumatic arrest, Practice management guideline

## Abstract

**Background:**

Emergency department thoracotomy (EDT) is a high-risk, salvage procedure performed during the resuscitation of critically injured patients who are in extremis or experience cardiac arrest immediately before or after arrival at the emergency department (ED) following trauma. Despite its potential to improve outcomes in selected patients, its indications and ideal clinical applications remain controversial due to significant risks of morbidity and mortality. In Saudi Arabia, trauma is responsible for approximately 20% of fatalities and is the leading cause of disability-adjusted life-years lost among males. However, there is limited research on EDT outcomes in this region.

**Methods:**

This retrospective cohort study was conducted at a tertiary academic trauma center in Riyadh with dedicated trauma and surgical services. Medical records from 2015 to 2023 were reviewed to identify adult patients who underwent EDT, using trauma team activations as a screening criterion. Data collected included demographics, mechanisms of injury, procedure indications, and timing.

**Results:**

During the study period, the center averaged 151 trauma activations per year, identifying 11 patients who underwent EDT. All were male, with a mean age of 35.64 ± 8.89 years. Blunt trauma was the cause in 64.6% of cases. Of these, 45.4% met the Eastern Association for the Surgery of Trauma (EAST) guideline recommendations for EDT, while 18.2% had contraindications. Traumatic arrest in the ED or prehospital settings was the most common indication 72.7%. Mean time between ED arrival to procedure initiation was 25.45 ± 15.85 min. One patient survived to discharge. Among 45 traumatic arrests screened, 4.44% lacked documentation for withholding EDT despite conditional guideline recommendations.

**Conclusions:**

Given the limited data available on EDT in the MENA region, our study offers important perspectives from a high-volume trauma center in KSA, where the rate of EDT was found to be very low. This low rate is likely due to the predominant injury patterns observed, presenting few candidates who might benefit from the procedure. At the same time, most patients who may have benefited based on guideline recommendations did undergo the procedure. These findings emphasize the need for heightened clinician awareness and systematic decision-making regarding EDT in trauma care. To enhance the generalizability of our findings and assess EDT’s utility in regional settings, further research involving larger multicenter registries is necessary in the country and the region.

## Introduction

EDT is a salvage procedure for resuscitation of critically injured patients who arrive in extremis or who have a cardiac arrest prior to or during presentation to the emergency department (ED) following a traumatic injury [1, 2]. It involves accessing the thoracic cavity through the ribs to identify the source of shock, potentially relieve cardiac tamponade, control intrathoracic or cardiac hemorrhage, perform open cardiac massage, and clamp the descending aorta to manage likely sub-diaphragmatic hemorrhage [3]. Its performance is associated with a high likelihood of patient morbidity and mortality, and its optimal indications have remained controversial [4, 5]. Further complicating the procedure is the high rate of operator exposure following an EDT, estimated to be 7–17% [6, 7]. In 2015, EAST issued guidelines to help determine the selection of patients for EDT. These guidelines were based on a comprehensive analysis of 72 studies involving 10,238 patients. They established indications and relative contraindications for performing the procedure in patients suffering from a traumatic cardiac arrest at presentation or just before reaching the ED, based on the mechanism of injury and presence or lack of signs of life [8]. 

Considering that traumatic injuries make up 20% of all deaths in the Kingdom of Saudi Arabia (KSA), they are the leading cause of disability-adjusted life years (DALYs) lost among males in high-income Arab countries. They have also become a recent focus of public health interventions, complementing nationwide efforts to prevent their occurrence in the first place. Understandably, improving survival and function after traumatic injuries remains an area of paramount interest and active research [9–12]. However, the research into EDT outcomes in KSA is lacking, with no publication on the subject made available.

Despite the availability of practice management guidelines, the decision to perform EDT often varies across institutions and clinicians, influenced by systemic impediments such as limited exposure to the procedure, variability in training, resource availability, and concerns about futility [13–15]. Understanding not only the outcomes of patients who undergo EDT but also the reasons why eligible patients may not receive the procedure is essential to identify barriers to guideline adherence and opportunities for improving trauma care. Therefore, in this study we aimed to estimate the survival rate of patients who underwent EDT at a tertiary trauma center in Saudi Arabia, while also comparing their indications with patients who presented with traumatic arrest but did not undergo EDT.

## Methods

This retrospective cohort study was conducted at a large academic tertiary care center in Riyadh, Saudi Arabia. Our ED manages approximately 100,000 adult visits annually, delivering comprehensive trauma care with support from relevant surgical subspecialties.

The study included all adult patients who underwent EDT between Jan 2016-Dec 2023. Cases were identified using trauma team activation (TTA) as a screening criterion, between 2015 and 2020 TTA were not protocolized, and lacked patient specific data for activation.

A secondary objective was to evaluate patients presenting with traumatic cardiac arrest who were assessed by the trauma team between 2020 and 2023. This analysis aimed to quantify adherence to established recommendations for performing EDT. Cases meeting EAST indications for EDT but in which the procedure was not performed were reviewed in consensus by a panel of four emergency physicians to assess the justification for withholding the intervention.

Statistical analysis was done using SPSS (IBM Co., Armonk, NY, USA). Numerical data were presented as the mean and standard deviation (SD), and the results were analyzed using the ANOVA (F) test. Categorical data were presented as the frequency and percentages.

All data were collected after obtaining the approval of the Institutional Review Board of King Saud University (reference no. E-22-6921). The IRB deemed consent not applicable due to the retrospective nature of data extracted from the hospital health information system.

## Results

Between 2015 and 2023, our center averaged 146.3 (SD ± 41.6) trauma code activations annually. Of these, (*n* = 45) involved traumatic cardiac arrests attended by the trauma team. 11 patients received EDT, including three patients who did not suffer a cardiac arrest. All EDT recipients were male, averaging 35.64 years (SD ± 8.89).

Regarding the nature of the trauma, 64.60% of the 11 patients who underwent EDT experienced blunt trauma (*n* = 7), while 18.20% suffered penetrating injuries (*n* = 2). The remaining 18.20% sustained a combination of blunt and penetrating trauma (*n* = 2). (Table 1) lists the identified injuries of these patients, as documented in their medical records. Injuries were detected during bedside examinations, Extended Focused Assessment with Sonography for Trauma (E-FAST), and/or during the EDT procedure.

**Table 1:** Injury location among the studied patients (*n* = 11)

Case, year performed	Mechanism of injury	Key findings/indication	(TI) min*	Outcome
1(2022)	Penetrating	Multiple stab wounds	5	Died
2(2018)	Penetrating	Hemopericardium (aortic injury)	11	Survived
3(2023)	Blunt	Unidentified	42	Died
4(2021)	Blunt	Rib fractures, cardiac laceration	16	Died
5(2023)	Combined	Massive thoraco-abdominal trauma	35	Died
6(2023)	Combined	Hemopericardium (on echo only)	10	Died
7(2021)	Blunt	Hemothorax, pelvic injury	24	Died
8(2022)	Blunt	Traumatic brain injury, Multiple thoracic/neck injuries, pelvic instability	37	Died
9(2023)	Blunt	Hemoperitoneum, multiple long bone fractures	26	Died
10(2023)	Blunt	Positive FAST, hypotension.	Immediate	Died
11(2023)	Blunt	Head injury, Hemothorax, positive FAST	15	Died

*TI: From loss of vital signs to initiation of procedure

*FAST* Focused Assessment with Sonography in Trauma

When comparing our patients who underwent EDT to the EAST guideline recommendations, 45.35% met the guidelines for performing EDT, while 18.20% met the guidelines against it. Additionally, 27.30% (*n* = 3) presented with a palpable pulse but had other clinical indicators supporting EDT (Table 2). At the trauma scene, 90.90% of our patients demonstrated at least one indicator of life, including pupillary response, spontaneous breathing, palpable pulses, measurable blood pressure, extremity movement, or cardiac electrical activity. During the ED assessment, 63.60% demonstrated signs of life. Our patients had various indications for EDT, with traumatic arrest being the most common (72.70%). The other three patients (27.30%) with a palpable pulse had indications of refractory hypotension, hemopericardium identified by E-FAST, and massive hemothorax identified by E-FAST.

**Table 2:** EAST indications among patients who underwent EDT

EAST guidelines recommendations	*N* = 11	%
Not pulseless; not included in the EAST guidelines	3	27.3
Patients presenting pulseless to the ED with signs of life after penetrating thoracic injury, EDT is strongly recommended	1	9.1
Patients presenting pulseless to the ED without signs of life after penetrating thoracic injury, EDT is conditionally recommended	2	18.2
Patients presenting pulseless to the ED with signs of life after blunt injury, EDT is conditionally recommended	3	27.3
Patients presenting pulseless to the ED without signs of life after blunt injury, it is conditionally recommended that EDT not be performed.	2	18.2

*EAST* Eastern Association for the Surgery of Trauma, EDT: Emergency department thoracotomy

Notably, the duration from arrival to the ED to initiating the EDT procedure at our center ranged from 5 to 56 min, with a mean duration of 25.45 ± 15.85 min. Of the 11 patients who underwent EDT, survival outcomes varied by mechanism of injury. All nine patients with blunt or combined trauma died before hospital admission. In contrast, 1 of 2 patients with penetrating trauma (50%) survived to hospital discharge with intact neurological function. This survivor also arrived with signs of life and a pulse.

Between 2020 and 2023, (*n* = 35) patients in traumatic cardiac arrest were identified who presented to our ED, underwent trauma team assessment, and did not receive an EDT (Table 3). Among these, 28.57% (*n* = 10) had a conditional recommendation for EDT according to the EAST guidelines. Upon reviewing individual cases, we found that in 8 of these 10 cases, there was reasonable justification for withholding the procedure, such as suspected severe head injury and/or multisystem trauma. However, in 2 cases, no clear rationale was documented in the electronic medical records despite the patients meeting the criteria.

**Table 3:** EAST indications among patients who did not undergo EDT

EAST guidelines recommendations	*N* = 35	%
Patients presenting pulseless to the ED without signs of life after blunt injury, it is conditionally recommended that EDT not be performed.	25	71.43
Patients presenting pulseless to the ED with signs of life after blunt injury, EDT is conditionally recommended	10	28.57

*EAST* Eastern Association for the Surgery of Trauma, EDT: Emergency department thoracotomy

## Discussion

In our retrospective single-center study spanning eight years, we observed 11 patients who underwent EDT. Blunt mechanism through motor vehicle collision (MVC) was the primary cause of injuries requiring it, followed by fall from a height. Notably, the study cohort exclusively consisted of males, reflecting the predominantly male driver population until recent years [16]. These findings are consistent with the existing literature, where male patients represent the majority undergoing EDT [3, 10]. 

Notably, our cohort’s age was within the reported favorable range for neurologically intact survival, with the literature demonstrating increasing age as an independent risk factor for mortality, with age over 60 being associated less than 10% survival in all-comers [17]. 

In contrast, our cohort injury patterns were mostly less favorable to survive after an EDT, with the majority having a blunt or a mixed injury pattern, with only one patient having a traumatic arrest with an exclusive penetrating injury. Penetrating chest injuries have demonstrated improved survival rates compared to other types of injuries post EDT [13, 17]. With penetrating stab wounds having better survival rates as compared to those with gunshot wounds [15]. 

Our study focused exclusively on adult patients undergoing EDT due to the absence of a dedicated pediatric trauma team, and therefore, pediatric EDT cases were not screened for. To note, our institutional cut-off for adult patients is > 14 years, with the youngest patient in our cohort being 25 years old. The literature regarding pediatric trauma suggests that the outcomes of pediatric EDTs are comparable to those of adult EDTs [18]. 

Over eight years, with an average of 151 trauma activations per year, only 11 cases underwent EDT, highlighting the rarity of this intervention within our cohort. Similarly, Hansen et al. reported the infrequency of EDTs, with approximately 952 performed annually across the United States, primarily in urban, high-volume trauma centers [13]. 

Among our cohort, the lone survivor had a penetrating thoracic injury and arrived with a pulse, both factors consistently linked to improved survival after EDT and aligned with EAST guidelines. While prior studies suggest that thoracotomies performed in the operating room yield superior outcomes due to better resources and monitoring, our data cannot determine whether this would have altered the result in this case [5, 19]. Instead, the case underscores the importance of timely recognition and intervention in appropriately selected patients.

At our institution, we observed a wide range of time intervals between 5 and 56 min between the patient’s arrival at the ED and initiating EDT. Several variables may have contributed to the delay in performing EDT, including the type and severity of the injury, the necessity for a comprehensive evaluation, logistical hurdles, the intricate decision-making process inherent to trauma scenarios, and, potentially, unfamiliarity with performing this procedure. Timely initiation of thoracotomy upon ED arrival is imperative for optimizing patient outcomes and increasing the likelihood of survival [20]. Similarly, prompt surgical intervention and coordinated resuscitation efforts are paramount for achieving favorable patient outcomes in cases necessitating resuscitative thoracotomy [21]. 

Our findings suggest that EDT is performed in a conservative manner in our institution with 10 patients not having undergone EDTs despite meeting conditional recommendations. The literature has demonstrated that performing EDT outside established guidelines is associated with poor outcomes, emphasizing the importance of adhering to protocols [17]. However, a more liberal approach may be beneficial, as Segalini et al. demonstrated an increase in overall survival rate from 10 to 23.50% following a more liberal policy, yet guidelines-driven, with no long-term neurological sequelae in survivors. Suggesting balancing broader applications and careful patient selection to achieve the best outcomes [22]. 

The literature on hesitancy in performing the procedure at capable centers highlights several contributing factors, including inadequate training and lack of experience, which can be addressed through simulation and cadaveric training. Other factors include resource limitations and perceived futility or uncertainty from inconsistent guideline recommendations [14, 23, 24]. 

Our analysis of patients who met conditional EAST criteria but did not undergo EDT provides insight into potential systemic barriers to implementation. While most of these cases had reasonable clinical justification for withholding the procedure, two lacked documented rationale despite guideline support. This highlights the value of comparing performed versus non-performed EDT cases, as such comparisons can reveal both appropriate conservatism and possible missed opportunities. In our setting, factors such as limited procedural exposure, variability in physician confidence, and perceived futility may have contributed to hesitancy. Recognizing these systemic impediments is critical to designing targeted interventions—such as structured training, clearer documentation protocols, and multidisciplinary review processes—to improve adherence to evidence-based guidelines.

This study has several limitations. First, it was conducted at a single center, which may limit the generalizability of the findings to other populations. The retrospective design also introduces inherent biases, including reliance on documentation quality and the inability to control for unmeasured confounders. Additionally, EDTs were not coded as individual procedures in the electronic medical records and had to be identified through manual review of trauma team activations, potentially leading to missed cases. Furthermore, the screening criteria likely under-identified cases of traumatic cardiac arrest, particularly when the attending emergency physician deemed it futile to activate trauma services. Due to limitations in institutional data entry practices, we were unable to capture key variables such as injury severity scores and anatomical injury patterns, which restricts comparability with other published literature.

Another limitation is the lack of a standardized approach to assessing adherence to guidelines. While we reviewed cases against EAST recommendations, the absence of a formal institutional protocol may have contributed to variability in practice and documentation. Prehospital care variability is another factor that was not captured but could have influenced both patient eligibility for EDT and subsequent outcomes, especially in a region where prehospital trauma systems are still evolving. Operator experience and training were not systematically assessed, yet these factors likely affected both the decision to perform EDT and procedural success. Lastly, using the time from ED arrival to EDT initiation as a proxy for timeliness may be suboptimal, as some patients only met indications later during their ED stay.

## Conclusion

Given the limited data available on EDT in the MENA region, our study offers important perspectives from a high-volume trauma center in KSA, where the rate of EDT was found to be very low. This low rate is likely due to the predominant injury patterns observed, presenting few candidates who might benefit from the procedure. At the same time, most patients who may have benefited based on guideline recommendations did undergo the procedure.These findings emphasize the need for heightened clinician awareness and systematic decision-making regarding EDT in trauma care. To enhance the generalizability of our findings and assess EDT’s utility in regional settings, further research involving larger multicenter registries is necessary in the country and the region.

## Data Availability

The entire de-identified dataset, data dictionary and analytic code for this investigation are available upon request, from the date of article publication by contacting Doctor Alageel, MD, FRCPP at email: moalageel@ksu.edu.sa.
